# Sequential inspiratory muscle exercise-noninvasive positive pressure ventilation alleviates oxidative stress in COPD by mediating SOCS5/JAK2/STAT3 pathway

**DOI:** 10.1186/s12890-023-02656-5

**Published:** 2023-10-12

**Authors:** Yirou Lei, Jiaying He, Fang Hu, Hao Zhu, Jing Gu, Lijuan Tang, Man Luo

**Affiliations:** 1 The First Affiliated Hospital of Hunan Normal University (Hunan Provincial People’s Hospital), Changsha City, 410016 Hunan Province P.R. China; 2 Department of Respiratory Medicine, The First Affiliated Hospital of Hunan Normal University (Hunan Provincial People’s Hospital), No. 89 Guhan Road, Furong District, Changsha City, 410016 Hunan Province P.R. China

**Keywords:** Chronic obstructive pulmonary disease, Noninvasive positive pressure ventilation, Inspiratory muscle training, Oxygen therapy, Oxidative stress

## Abstract

**Background:**

Pulmonary rehabilitation training is of great significance for the prognosis of chronic obstructive pulmonary disease (COPD) patients. The purpose of this study was to investigate the therapeutic effect and pathway of a new sequential noninvasive positive pressure ventilation (NIPPV) + inspiratory muscle training (IMT) therapy.

**Methods:**

A total of 100 COPD patients were enrolled and randomly divided into oxygen therapy (OT), NIPPV, IMT and sequential (NIPPV + IMT) group. Lung function, exercise endurance, quality of life, and dyspnea symptoms were examined and recorded. Then, reactive oxygen species (ROS), malonaldehyde (MDA), superoxide dismutase (SOD) and glutathione (GSH) levels were detected by enzyme-linked immunoassay, and suppressor of cytokine signaling 5 (SOCS5)/janus kinase 2 (JAK2)/signal transducer and activator of transcription 3 (STAT3) pathway expression changes were detected by quantitative real time-polymerase chain reaction (qRT-PCR) and western blot. A mouse model of COPD was then established to further verify the effects of SOCS5/JAK2/STAT3 pathways on lung function and oxidative stress.

**Results:**

After 8 weeks of treatment, NIPPV, IMT or sequential (NIPPV + IMT) significantly improved exercise endurance, quality of life and dyspnea, reduced oxidative stress, promoted SOCS5 expression and inhibited the activation of JAK2/STAT3 pathway, and no significant effect was observed on lung function of COPD patients. Notably, sequential (NIPPV + IMT) showed better therapeutic outcomes than either IMT or NIPPV alone. Moreover, results at the animal level showed that overexpression of SOCS5 significantly reduced pulmonary inflammatory infiltration, pathological changes and oxidative stress levels in COPD mice, enhanced lung function, and inhibited the activation of JAK2/STAT3 pathway.

**Conclusion:**

Our results elucidated that sequential (NIPPV + IMT) significantly relieved COPD development by regulating SOCS5/JAK2/STAT3 signaling-mediated oxidative stress.

**Supplementary Information:**

The online version contains supplementary material available at 10.1186/s12890-023-02656-5.

## Introduction

Chronic obstructive pulmonary disease (COPD) is a common progressive respiratory disease characterized by persistent airflow restriction, and is a significant cause of disease death worldwide [1]. The latest epidemiological survey in China shows that the prevalence of COPD among people over 40 years old increased from 8.2% to 2008 to 13.7% in 2015, which is also related to the aging population [2]. Risk factors such as smoking, environmental pollution, occupational dust and chemical exposure, infection, and genetic factors can cause immune and inflammatory disorders, redox imbalances, and airway and vascular remodeling, leading to COPD occurrence and deterioration [3, 4]. However, the exactly pathogenesis is still not fully understood. Thus, it is of great significance to elucidate the pathogenesis of COPD for searching new effective therapeutic approaches.

Pulmonary rehabilitation is an individualized treatment strategy, which aims to improve the physical and psychological conditions of COPD patients and promote their long-term healthy behaviors through exercise training, self-control management and behavioral intervention [5]. Due to early airway obstruction, heavy respiratory load and low respiratory efficiency, respiratory work increases and respiratory muscle strength decreases, which leads to respiratory muscle dysfunction, resulting in dyspnea and decreased exercise tolerance in COPD patients [6, 7]. Although the current intervention of respiratory muscle function rehabilitation such as inspiratory muscle training (IMT) and calisthenics can partially improve the health status of COPD patients [8, 9]. However, training for specific respiratory muscle groups is rare. Noninvasive positive pressure ventilation (NIPPV) is a controversial form of therapy for COPD patients [10]. Clinical study showed that NIPPV could improve the survival in hypercapnia, stable COPD patients [11]. However, there was few COPD patients who receive acute or continuous NIPPV are at potential risk for serious complications such as pulmonary barotrauma and bleeding [12, 13]. On this basis, we proposed for the first time the rehabilitation strategy of sequential NIPPV + IMT that make the inspiratory muscles get a full rest after a certain intensity load exercise. However, the clinical efficacy of this strategy are still unclear.

The imbalance of oxidation/antioxidant system is one of driving mechanism in pathogenesis of COPD. For decades, there were a great number of reports confirmed that enhancing endogenous antioxidant stress is an effective strategy for treating COPD [14, 15]. Suppressor of cytokine signaling 5 (SOCS5) is a main member of SOCS family, which are known to be cytokine-inducible negative regulators of cytokine signaling, also called as signal transducer and activator of transcription (STAT)-induced STAT inhibitor (SSI) protein family. A recent finding revealed that the maintain of SOCS5 could alleviate lipopolysaccharide-induced oxidative stress and the release of pro-cytokine factors to attenuate myocardial injury [16]. More importantly, Diao et al’s study also confirmed that downregulation of SOCS5 targeted by miR-132 could aggravate the inflammatory responses in COPD pathophysiology [17]. Janus kinase 2 (JAK2)/STAT3 signaling as the main intracellular signaling chain that is a key downstream pathway of SOCS5, plays an important role in cell proliferation, apoptosis, oxidative stress, and immune response [18, 19]. For example, Piao et al’s report revealed that inhibition of IL-6/JAK2/STAT3 signaling could efficiency alleviate inflammatory responses and oxidative stress, thereby relieving pancreatitis-induced lung injury [20]. Therefore, it could be considered that SOCS5 might mediate JAK2/STAT3 pathway to regulate imbalance of oxidation/antioxidant processes in COPD.

In the present work, we aimed to explore the clinical therapeutic effect of sequential NIPPV + IMT in COPD patients and its association with SOCS5/JAK2/STAT3 signaling pathway, as well as the biological roles of SOCS5/JAK2/STAT3 signaling in oxidative stress damage in COPD mice model. Thus, we proposed a hypothesis that sequential NIPPV + IMT might improve exercise tolerance, quality of life, dyspnea and oxidative stress in COPD patients by regulating SOCS5/JAK2/STAT3 signaling pathway.

## Methods

### Clinical subject information and ethical statements

All clinical trials have been approved by The First Affiliated Hospital of Hunan Normal University (Hunan Provincial People’s Hospital, 2023 No. 180), and all subjects have signed the statement of interest before participating. In the present work, a total of 100 patients with COPD were included in this study, all of whom were diagnosed after medical history, physical examination, pulmonary function examination and X-ray examination according to the COPD diagnostic criteria specified by GOLD. All patients received conventional treatment and did not need to stop any of the conventional treatment drugs before examination.

The inclusion criteria were: (1) age range from 40 to 80 years old; (2) severe (30% pre ≤ FEV1% < 50% pre) or extremely severe (FEV1% < 30% pre or FEV1% < 50% pre with chronic respiratory failure) COPD patients; (3) difficulty breathing; (4) there was no acute exacerbation in the last 4 weeks. The exclusion criteria were as follows: (1) those who smoked more than 10 cigarettes a day; (2) BMI > 40 kg/m^2^; (3) patients with cardiac hemodynamic instability; (4) patients with other respiratory diseases, neuromuscular diseases and serious cerebrovascular accident sequelae.

Observation was terminated if the following conditions occurred: (1) the patient died; (2) refuse to participate in the IMT or NIPPV therapy or oxygen therapy (OT) in this study; (3) occur pneumothorax, the patient’s ability to clear secretions from oropharynx and upper respiratory tract is decreased, the patient can not normally use the nose mask, can no longer tolerate non-invasive ventilation, acute cardiovascular and cerebrovascular events, severe liver insufficiency, severe renal insufficiency, newly diagnosed malignant tumor or mental abnormality.

### Experimental grouping and treatment

**Fig. 1:**
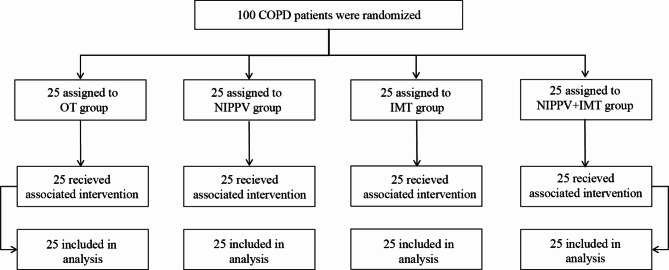
Flow chart of the study

All subjects were randomly divided into 4 groups in equal proportions using a random number generator: (1) OT group (male 21, female 4), NIPPV group (male 23, female 2), IMT group (male 20, female 5) and NIPPV + IMT (male 21, female 4). Randomization was performed using opaque sealed envelopes that were opened during screening visits. All subjects received OT for at least 8 h per day with SPO_2_ > 90% and oxygen flow < 6 L/min. When NIPPV was treated, the initial EPAP was set at 4 cm H_2_O, and IPAP was gradually adjusted until the patient felt most comfortable and IPAP-EPAP was > 10 cm H_2_O. Oxygen absorption concentration (FiO_2_) to maintain blood oxygen saturation ≥ 90%, oxygen flow 4 ~ 6 L/min. The standby ventilation frequency is set to 14 times per min, and the suction rise time is set to 0.5-2 s. The duration of treatment was not less than 4 h in the daytime and continued at night. When IMT treatment, the patient was given a Power breath K5 inspiratory muscle trainer (Powerbreath Technologies Ltd, Birmingham, UK). The training intensity started at 40% PImax for 1 week, and then gradually increased by 10% until it reached 80% PImax. Exercise for 30 min twice a day. In NIPPT + IMT group, patients received IMT training for 30 min/session and followed by NIPPV for 2 h, twice a day. All patients were treated for 8 weeks. The flow chart was provided as Fig. 1.

### Lung function examination

The lung function of the patients was measured using the Jaeger pulmonary function instrument Spi rostik CompTete (Jaeger, Germany), according to the guidelines formulated by the European Respiratory Society (ERS) and the American Thoracic Society (ATS) [21].

### Exercise tolerance test

The exercise tolerance was measured by the 6-min walking test (6MWT) according to ATS guidelines for the Application of 6MWT [22]. In short, COPD patients walked from the starting point to the stopping point at the end of the flat indoor corridor with a reentry length of 30 m, the distance was measured (Supplementary Table 1). Recording the basic pulse, blood pressure, heart rate and pulse oxygen saturation at the before and after the test. Borg score was applied to assess basic dyspnea and fatigue index (Supplementary Table 2).

### Score of health-related quality

The severe respiratory insufficiency (SRI) questionnaire developed and compiled by Professor Windisch’s team was used to evaluate the quality of life of the patients in this study [23]. The scale is completed by the subject independently, and the investigator is not allowed to make any suggestive reminders. The SRI scale consists of seven parts: physical functioning (SRI-PF), respiratory complaints (SRI-RC), attendant symptoms and sleep (SRI-AS), social relationships (SRI-SR), social functioning (SRI-SF), anxiety (SRI-AX) and psychological well-being (SRI-WB). After the survey, the score of each part and the total score of the whole questionnaire were calculated by the investigator through conversion and weighting. The score ranges from 0 to 100, and the score was positively correlated with the health status of patients.

### Dyspnea score

Modified medical research council (MRC) scale was used to evaluate the degree of dyspnea of patients [24](Table 1), in which 0 was mild. A score of 1 is moderate; A score of 2–4 is considered severe.

**Table 1:** MRC scale

MRC score	Characteristics
0	Breathing is usually not difficult except during strenuous exercise
1	Shortness of breath when walking steeply or uphill
2	Walking on flat ground is slower than peers due to shortness of breath
3	Shortness of breath comes after walking 100 m or a few min on the flat
4	Obvious shortness of breath, can not leave the house, dressing, stripping have shortness of breath

MRC: Medical research council

### Oxidative stress detection

By referencing the previous report, 5 mL blood samples were taken by fasting venipuncture into the EDTA vacuum collection vessel under sterile conditions [25]. After centrifugation, the serum sample was collected to examine the levels of reactive oxygen species (ROS, ab113851, Abcam, Cambridge, MA, USA), malondialdehyde (MDA, ab118970, Abcam) and activities of superoxide dismutase (SOD, ab80946, Abcam) and glutathione (GSH, ab239727, Abcam) according to the kit instructions.

### Western blot

The experimental procedure was performed by previous described [26]. The proteins were isolated with RIPA and the cut gels were transferred to the PVDF membrane (Millipore, MA, USA). Then, membranes were incubated overnight with antibodies against SOCS5 (PA5-21600, Thermo Fisher Scientific), JAK2 (ab108596, Abcam), p-JAK2 (ab32101, Abcam), STAT3 (ab119352, Abcam), p-STAT3 (ab32143, Abcam), and GAPDH (ab8245, Abcam). After washed with TBST, membranes were then incubated with secondary antibody (ab7090, Abcam) for 60 min. The membranes were visualized and imaged by GEL imaging system (Bio-Rad, CA, USA). The bands were quantified by Image J (National Institutes of Health, MD, USA).

### Establishment of COPD mouse model

All animal experiments were performed in accordance with the animal ethics approval of The First Affiliated Hospital of Hunan Normal University (Hunan Provincial People’s Hospital, 2021 No. 43). C57BL/6 male mice (6–8 weeks) were purchased from SLAC Laboratory (Shanghai, China) and kept in an animal facility at 21 ~ 24 °C for 12/12 h with light and dark cycles and free access to water and food. Mice were randomly divided into Air + pc-NC group, Cigarette smoke (CS) + pc-NC group and CS + pc-SOCS5 group (n = 6/per group). In brief, mice were exposed to room air or CS of five cigarettes (Furongwang, HUNAN Tobacco Industrial Co., Ltd. China) four times each day for 30 min/time, 5 days/weeks for up to 24 weeks. The lentivirus-packed SOCS5 overexpressing vector (pc-SOCS5) and empty vector (pc-NC) assembled by GenePharma (Shanghai, China) were intranasally injected into the mice once every 2 weeks (2 × 10^7^ TU in 50 µL). The lung function of mice in each group were examined as the previous described [26]. After last CS/air exposure, the mice were euthanized by intraperitoneal injection of an overdose of pentobarbital sodium (800 mg/kg) on another day and pathological analysis was performed.

### H&E staining

As previous described [26], H&E staining was conducted to assess pathologic changes. The lung tissues of each group were collected and fixed with 4% buffer formalin solution and embedded in paraffin and then cut into 4 μm thick slices. Then, the slices were subjected to H&E staining by using H&E staining kit (Beyotime, China) according to instruction protocol. Finally, the slices were photographed under an optical microscope (Olympus, China).

### Statistical analysis

Statistical data was analyzed by SPSS 19.0 (IBM, NY, USA) and expressed as means ± SD. The differences between-group differences and multi-group comparisons were determined using Student’s *t* test and one-way ANOVA followed by Tukey’s post hoc tset, respectively. The *p* values less than 0.05 were considered significant.

## Results

### Effect of sequential NIPPV + IMT intervention on pulmonary function in patients with COPD

**Fig. 2:**
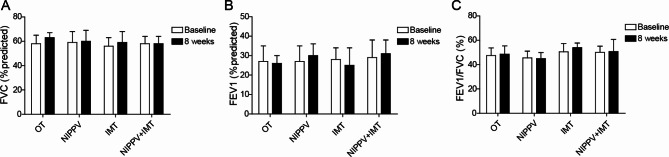
Effect of sequential NIPPV + IMT intervention on pulmonary function in patients with COPD. A total of 100 COPD patients were enrolled and randomly divided into OT group, NIPPV group, IMT group and NIPPV + IMT group. After 8 weeks of treatment, the lung function related indexes including FVC(A), FEV1(B) and FEV1/FVC(C) were measured by Jaeger pulmonary function instrument. All indices were determined at least three times

As shown in Table 2, patients in each group had no significant differences in age, BMI, lung function, respiratory muscle strength, blood gas analysis, exercise tolerance, dyspnea and quality of life scores. After 8 weeks of treatment, lung function was measured in each group. Compared with before intervention, IMT, NIPPV and sequential NIPPV + IMT had no significant effect on various pulmonary function indexes FVC, FEV1 and FEV1/FVC (Fig. 2A-C). These data indicated that sequential NIPPV + IMT intervention had no significant effect on pulmonary function in COPD patients.

**Table 2:** Comparison of clinical baseline data and clinical characteristics of patients

Group	OT(n = 25)	NIPPV(n = 25)	IMT(n = 25)	NIPPV + IMT(n = 25)
Age(years)	67.6 ± 7.6	68.2 ± 8.2	66.7 ± 8.9	67.2 ± 7.1
Sex(% male)	21(84%)	23(92%)	20(80%)	21(84%)
BMI(kg/m^2^)	22.3 ± 1.9	22.6 ± 2.4	23.1 ± 1.6	21.4 ± 2.2
Pack-years of smoking	47(33)	49(36)	45(32)	48(35)
FVC(%predicted)	57.6 ± 7.2	59.1 ± 8.5	55.6 ± 6.8	58.3 ± 5.9
FEV1(%predicted)	27.4 ± 8.3	26.9 ± 7.7	28.1 ± 6.2	29.3 ± 9.2
FEV1/FVC(%)	47.5 ± 6.2	45.5 ± 5.6	50.4 ± 6.8	50.1 ± 5.1
NIF(cm H_2_O)	-51.38 ± 8.2	-46.7 ± 5.8	-49.3 ± 6.6	-48.5 ± 8.3
PaCO_2_(mmHg)	44.2 ± 6.1	45.6 ± 5.3	44.8 ± 7.2	45.1 ± 6.5
PaO_2_(mmHg)	63.5 ± 8.9	66.4 ± 10.2	62.7 ± 9.7	65.8 ± 11.3
SaO_2_(%)	88.5 ± 8.2	91.4 ± 7.9	90.6 ± 8.5	89.7 ± 7.7
MIP(cm H_2_O)	-55.3 ± 13.2	-56.3 ± 11.8	-55.7 ± 10.8	-56.7 ± 12.2
MEP(cm H_2_O)	83.6 ± 16.3	86.5 ± 18.9	84.4 ± 17.5	87.4 ± 19.1
MRC dyspnea	3.5 ± 0.7	3.7 ± 0.9	3.4 ± 0.6	3.9 ± 0.8
6MWD(m)	254.5 ± 74.3	262.1 ± 69.1	271.5 ± 72.1	268.7 ± 67.7
SRI	42.1 ± 8.7	44.7 ± 9.3	45.3 ± 8.2	41.7 ± 6.7

FVC: Forced vital capacity; FEV1: Forced expiratory volume; NIF: Negative inspiratory force; PaCO_2_: Arterial carbon dioxide pressure; PaO_2_: Arterial oxygen pressure; SaO_2_: Arterial oxygen saturation; MIP: Maximal inspiratory pressure; MEP: Maximal expiratory pressure; MRC: Medical research council; 6MWD: 6 min walking distance; SRI: Severe respiratory insuffiency questionnaire

### Sequential NIPPV + IMT intervention enhanced the exercise tolerance of COPD patients

**Fig. 3:**
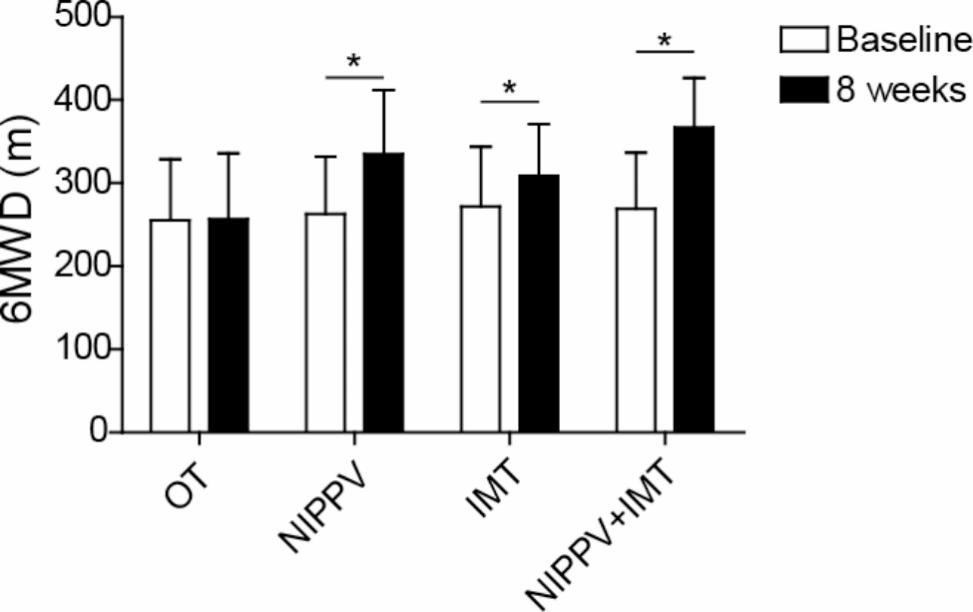
Sequential NIPPV + IMT intervention enhanced the exercise tolerance of COPD patients. A total of 100 COPD patients were enrolled and randomly divided into OT group, NIPPV group, IMT group and NIPPV + IMT group. After 8 weeks of treatment, 6MWT method was used to examine the exercise tolerance. All indices were determined at least three times. *P < 0.05

After 8 weeks of treatment, exercise tolerance was measured using the 6MWT method. As shown in Fig. 3, compared with before intervention, 6-min walking distance (6MWD) in NIPPV, IMT and sequential NIPPV + IMT groups was significantly increased. Moreover, sequential NIPPV + IMT treatment was significantly higher than that of IMT or NIPPV treatment (Fig. 3).

### Sequential NIPPV + IMT intervention significantly improved the quality of life in COPD patients

**Fig. 4:**
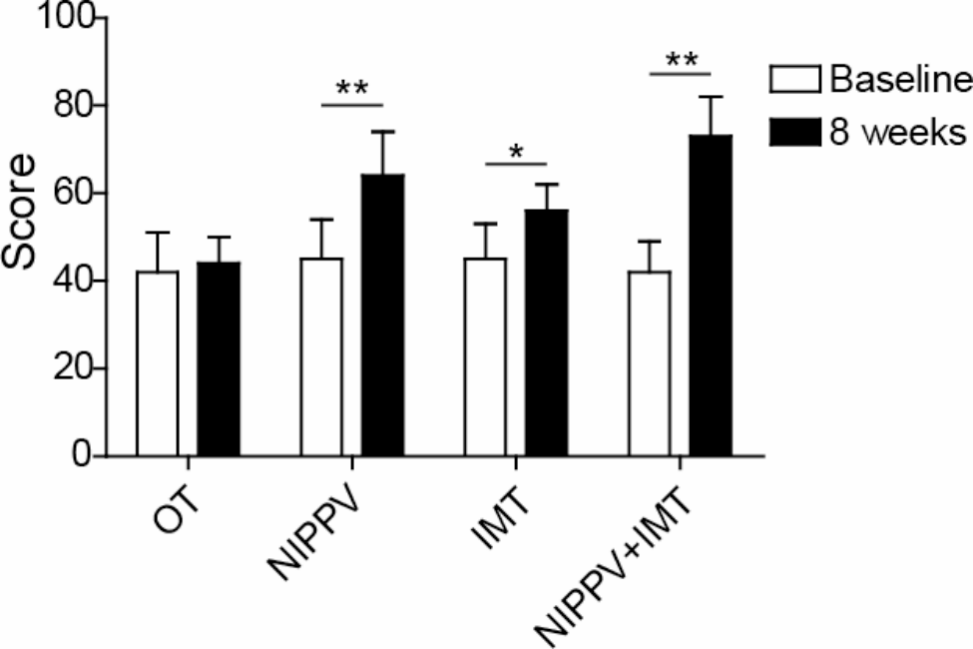
Sequential NIPPV + IMT intervention significantly improved the quality of life in COPD patients. A total of 100 COPD patients were enrolled and randomly divided into OT group, NIPPV group, IMT group and NIPPV + IMT group. After 8 weeks of treatment, SRI questionnaire was carried out to assess the quality of life of COPD patients. *P < 0.05, **P < 0.01

Compared to OT group, IMT, NIPPV and sequential NIPPV + IMT treatment remarkably enhanced the score of health-related quality (Fig. 4). In addition, compared to IMT and NIPPV groups, COPD patients in sequential NIPPV + IMT group had significantly better quality of life (Fig. 4).

### Sequential NIPPV + IMT treatment markedly alleviated dyspnea COPD patients

**Fig. 5:**
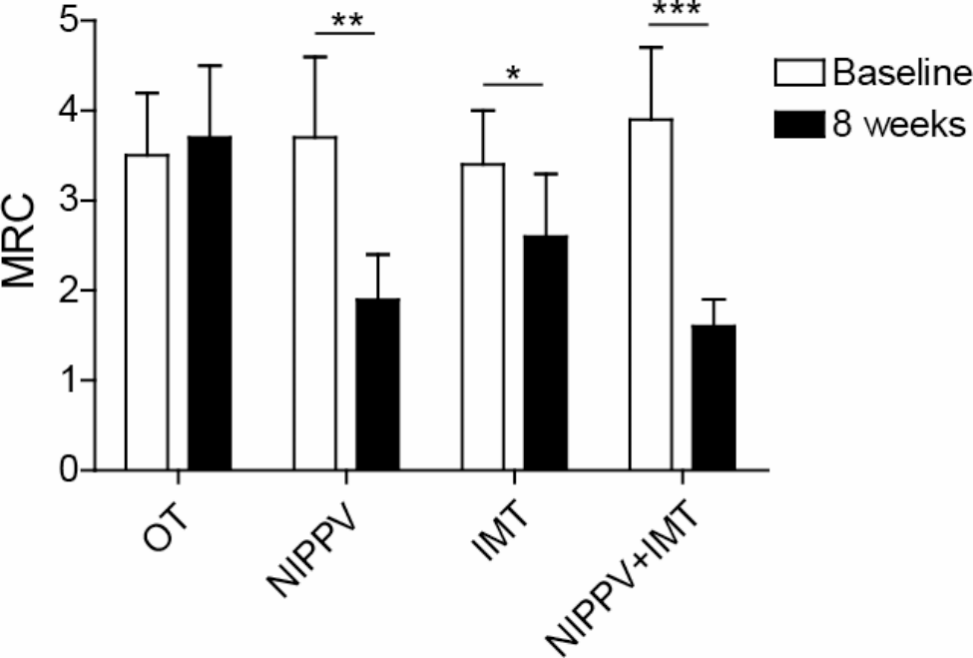
Sequential NIPPV + IMT treatment markedly alleviated dyspnea COPD patients. A total of 100 COPD patients were enrolled and randomly divided into OT group, NIPPV group, IMT group and NIPPV + IMT group. After 8 weeks of treatment, dyspnea in patients with COPD were evaluated by MRC scale. *P < 0.05, **P < 0.01, ***P < 0.001

The efficacy of sequential NIPPV + IMT treatment for dyspnea in patients with COPD were evaluated by MRC scale. After 8 weeks of treatment, the MRC score was strikingly reduced in patients in NIPPV, IMT and sequential NIPPV + IMT groups compared that in OT group (Fig. 5). Importantly, the MRC score in patients of sequential NIPPV + IMT group were also lower than that in patients of NIPPV and IMT groups (Fig. 5).

### Sequential NIPPV + IMT treatment relived the oxidative stress of COPD patients

**Fig. 6:**
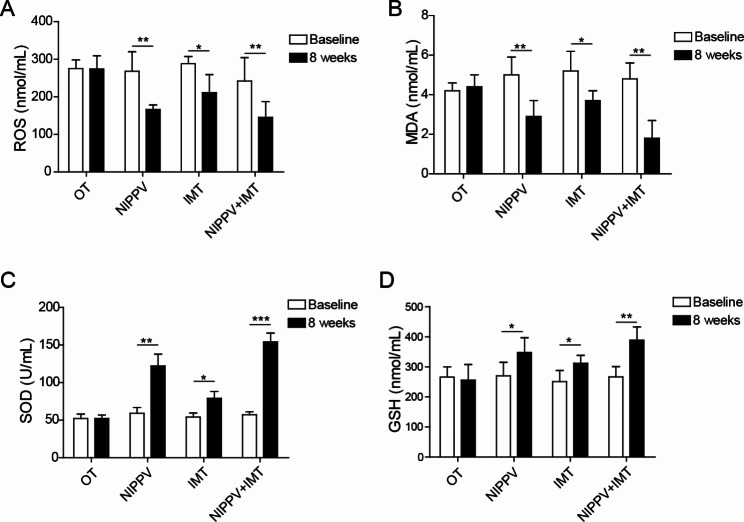
Sequential NIPPV + IMT treatment relived the oxidative stress of COPD patients. The serum samples were obtained from COPD patients in each groups, then the levels of ROS **(A)** and MDA **(B)** as well as the activities of SOD **(C)** and GSH **(D)** were tested by ELISA assay. All indices were determined at least three times. *P < 0.05, **P < 0.01, ***P < 0.001

By ELISA assay, we could observe that the ROS and MDA levels in patients of NIPPV, IMT and sequential NIPPV + IMT groups were obviously reduced, but the activities of SOD and GSH were upregulated, compared those in patients in OT group (Fig. 6A-D), suggesting NIPPV, IMT and sequential NIPPV + IMT treatment could alleviate the oxidative stress of COPD patients. Notably, the oxidation inhibition effect of sequential NIPPV + IMT treatment was significantly stronger than that of IMT or NIPPV treatment alone (Fig. 6A-D).

### Effects of sequential NIPPV + IMT treatment on SOCS5/JAK2/STAT3 signaling pathway

**Fig. 7:**
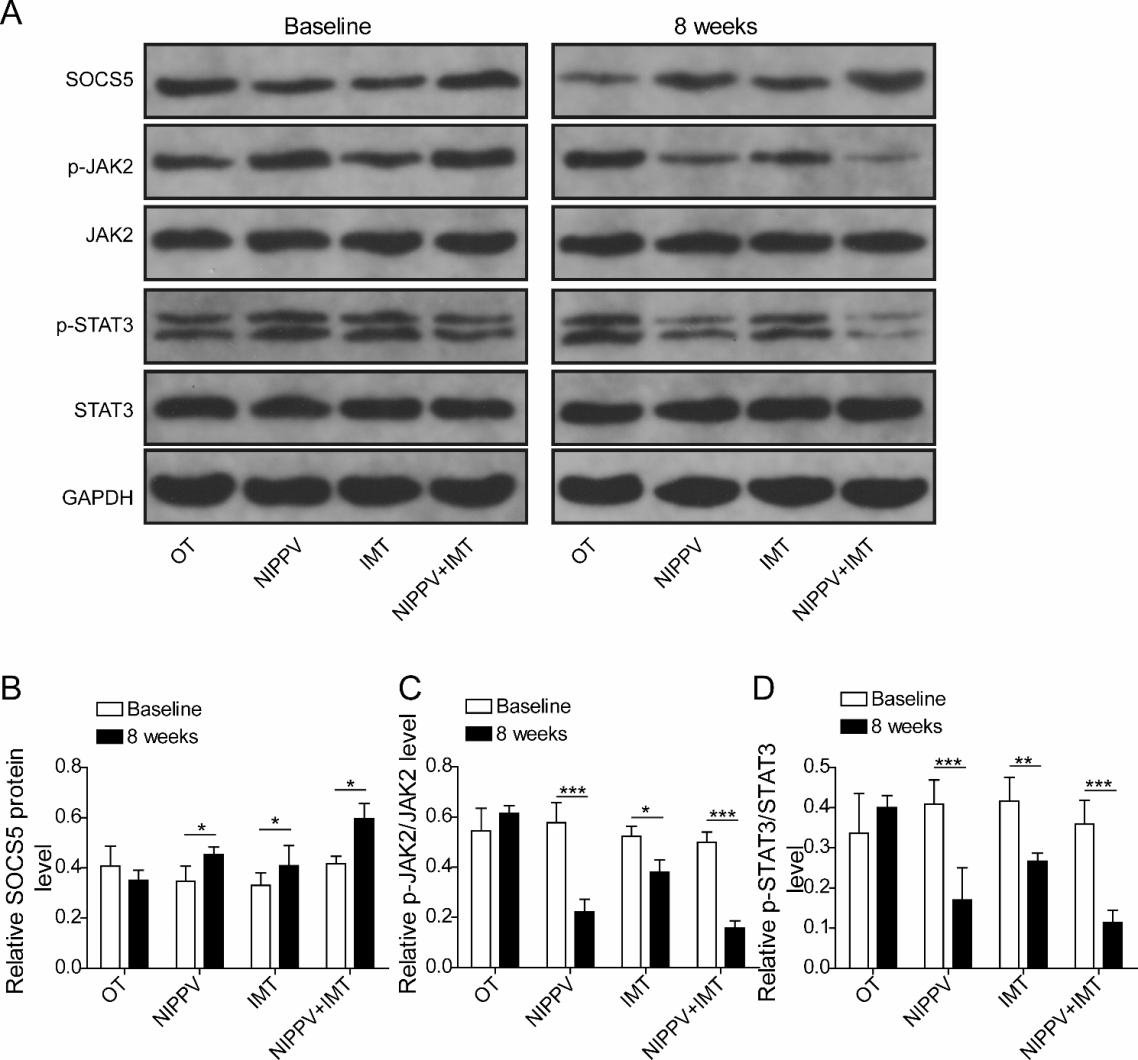
Effects of sequential NIPPV + IMT treatment on SOCS5/JAK2/STAT3 signaling pathway. **(A-D)** The protein levels of SOCS5, p-JAK2/JAK2, p-STAT3/STAT3 in serum samples of COPD patients were determined by western blot assay (The result shows the cut gel). All examinations were determined at least three times. *P < 0.05, **P < 0.01, ***P < 0.001

The results from western blot assay showed that the protein level of SOCS5 was upregulated, but the phosphorylation of JAK2 and STAT3 were reduced after NIPPV, IMT or sequential NIPPV + IMT treatment (Fig. 7A-D). Moreover, sequential NIPPV + IMT treatment showed a better intervention effect on the SOCS5/JAK2/STAT3 pathway than NIPPV or IMT treatment (Fig. 7A-D).

### SOCS5 suppressed COPD development by inactivating JAK2/STAT3 signaling

**Fig. 8:**
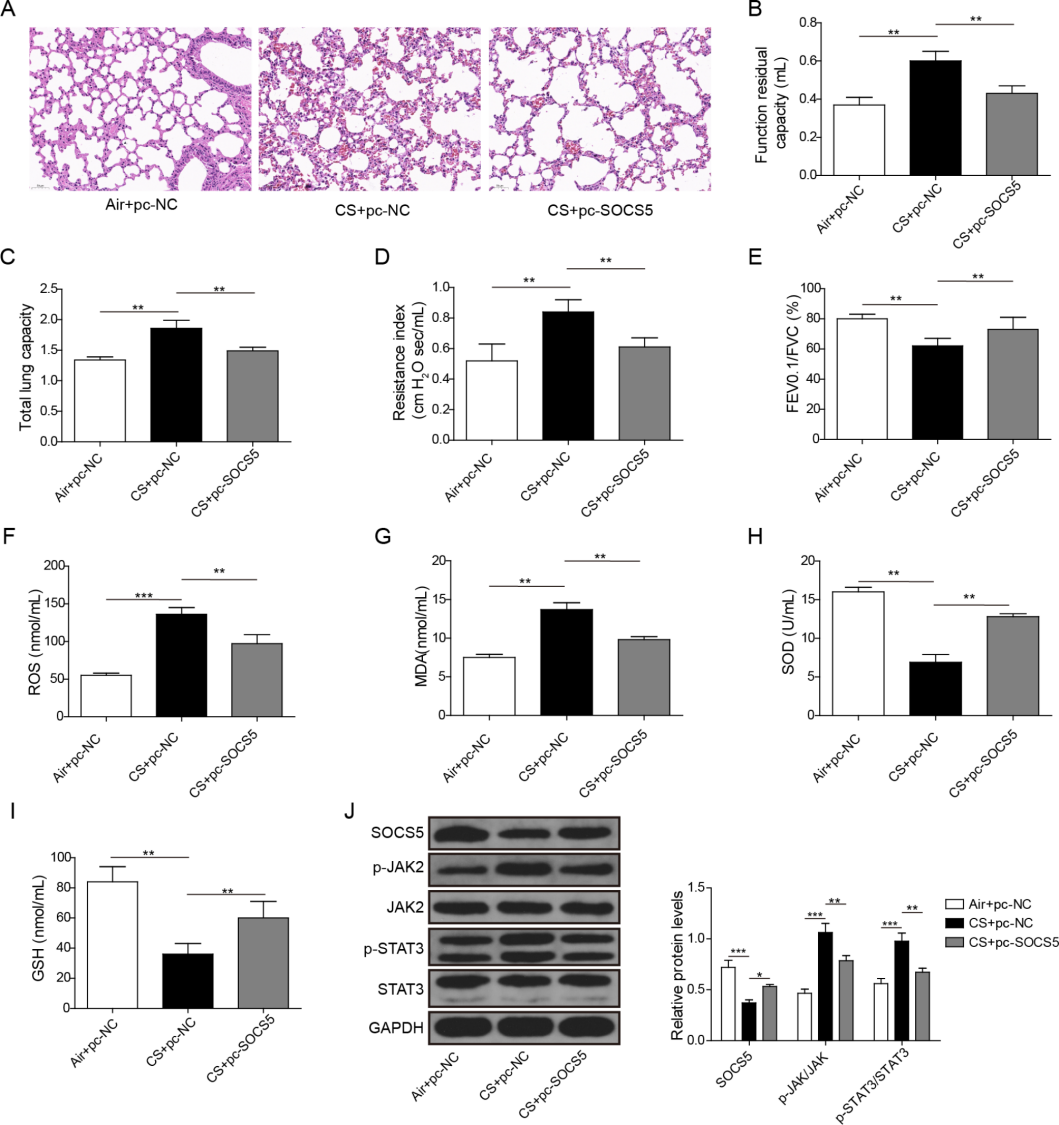
SOCS5 suppressed COPD development by inactivating JAK2/STAT3 signaling. C57BL/6 male mice were randomly divided into Air + pc-NC group, CS + pc-NC group and CS + pc-SOCS5 group (n = 6/per group). **(A)** H&E staining was carried out to assess the pathological changes. **(B-E)** Lung function index including Function residual capacity **(B)**, Total lung capacity **(C)**, Resistance index **(D)** and FEV0.1/FVC **(E)** were examined. **(F-I)** The levels of ROS **(F)**, MDA **(G)**, SOD **(H)** and GSH **(I)** were tested by ELISA assays. **(J)** The protein levels of SOCS5, p-JAK2/JAK2, p-STAT3/STAT3 were determined by western blot assay (The result shows the cut gel). *P < 0.05, **P < 0.01, ***P < 0.001

To further confirm the biological roles of SOCS5/JAK2/STAT3 signaling in COPD, we established COPD mouse model. H&E staining showed that the pulmonary parenchymal inflammatory cell infiltration, small airway thickening and alveolar cavity collapse were increased in the CS + pc-NC group compared to Air + pc-NC group, while this effect was alleviated by SOCS5 overexpression (Fig. 8A). Lung function measurement results showed that compared with the Air + pc-NC group, CS exposure markedly destroyed lung function, which was represented by increased functional residual capacity, total lung capacity and resistance index, and decreased FEV0.1/FVC. However, these effects were partially reversed after overexpression of SOCS5 (Fig. 8B-E). Next, the oxidative stress of mice were further examined. As shown in Fig. 8F-I, CS exposure significantly increased ROS and MDA level as well as downregualted the activities of SOD and GSH, and all of these effects were dramatically diminished by SOCS5 overexpression. Western blot analysis also presented that compared to Air + pc-NC group, the protein level of SOCS5 was reduced and the phosphorylation of JAK2 and STAT3 were enhanced in lung tissues of mice in CS + pc-NC group, and these protein levels were remarkably reversed by SOCS5 overexpression (Fig. 8J). Therefore, these results showed that SOCS5 improved COPD development by inactivating JAK2/STAT3 signaling pathway.

## Discussion

COPD is the most common chronic respiratory disease, with a high prevalence rate, high disability rate, high mortality characteristics, a serious threat to human health. According to a statistical report, the number of COPD patients worldwide reached 544.9 million in 2017, an increase of 39.8% since 1990 [27]. To data, there is still no effective treatment for COPD in clinic. With the continuous deterioration of COPD patients, it often involves multiple systems, resulting in respiratory muscle, limb muscle dysfunction and malnutrition, so that the emergence of year by year increased dyspnea, exercise tolerance and quality of life decreased, causing a great burden to society [28, 29]. Therefore, it is important to seek effective strategies to treat COPD and elucidate its pathways of action.

Currently, glucocorticoids, as the most common used anti-inflammatory drugs, are widely used in the treatment of acute COPD exacerbation and stable stage [30]. Although glucocorticoid therapy can improve lung function and reduce acute exacerbations, long-term use of glucocorticoid therapy does not prevent the decline of lung function, seriously affecting the quality of life of patients [31, 32]. In recent years, studies have shown that patients with different degrees of COPD can benefit from pulmonary Rehabilitation and exercise Training, including improved activity tolerance, reduced dyspnea and improved quality of life [33, 34]. For the majority of COPD patients, IMT is an effective stand-alone intervention that can be transitioned to aerobic and resistance training as part of a multidimensional respiratory rehabilitation training program for patients with dyspnea and exercise intolerance, as well as for patients who avoid activity due to fear of motor dyspnea [35]. Studies have shown that IMT therapy can increase type I and type II fibers of intercostal external muscles, induce remodeling of training muscles, and change the adaptive structure of respiratory muscles [8]. Beaumont et al. also uncovered that IMT effectively improves the strength and endurance of the inspiratory muscles, reduces breathing difficulties in activities of daily living, and improves walking distance and quality of life [33]. In addition, NIPPV was confirmed to significantly reduce the rate of endotracheal intubation, shorten the length of hospital stay, and avoid complications of invasive ventilation such as airway injury and ventilator-associated pneumonia [36]. Tang et al. highlighted that long term NIPPV treatment improved arterial blood gas, enhanced exercise endurance and quality of life, and reduced mortality in patients with COPD [37]. Similar results also found in Wiles et al’s work [10]. However, there is still some controversy about NIPPV’s role in COPD [38]. The failure of NIPPV treatment leads to the delay of endotracheal intubation time and treatment time, which increases the mortality of patients, which might relate to high CRP concentration, serum albumin, combined renal insufficiency, vomit aspiration [39–42]. Based on the above research status, we proposed for the first time a novel therapy with sequential NIPPV + IMT. Compared with IMT and NIPPV alone, sequential NIPPV + IMT treatment increased exercise endurance, improved quality of life and dyspnea symptoms in patients, and this process was associated with reduced levels of oxidative stress.

Oxidative stress directly damages airway and lung tissues, leads to protease/antiprotease imbalance, promotes inflammation, and plays an extremely important role in the occurrence and development of COPD [15]. Enhancing the antioxidant capacity of COPD patients and reducing oxidative stress damage are also considered to be the key to improve the progression of COPD [43]. Wang et al. revealed that aerobic exercise efficiency relieved emphysema and pulmonary fibrosis of COPD mice by suppressing oxidative stress and inflammation [44]. A pulmonary rehabilitation study showed that patients with COPD significantly reduced the oxidative stress capacity of exercise after 8 weeks of pulmonary rehabilitation training [45]. In our study, we demonstrated for the first time that both IMT and NIPPV treatment significantly reduced the levels of MDA and ROS in the serum of COPD patients, enhanced the activities of SOD and GSH, and significantly enhanced the antioxidant capacity of COPD patients. Importantly, sequential NIPPV + IMT treatment showed better therapeutic efficacy, suggesting that sequential NIPPV + IMT may be a novel way to improve COPD progression by intervening in oxidative stress.

Additionally, we also observed increased SOCS5 expression and inactivation of the JAK2/STAT3 pathway after IMT, NIPPV and sequential NIPPV + IMT therapy, suggesting that the SOCS5/JAK2/STAT3 signaling pathway might be involved in its mediated changes in oxidative stress levels and disease progression in COPD patients. SOCS family proteins are a class of negative regulators of JAK2-STAT3 signaling pathway, consisting of SOCS1-7, both containing Src-homology domian and a conserved C-termial domain [46]. Among them, SOCS5 is expressed in a variety of adult tissues, playing anti-inflammatory, anti-tumor, anti-oxidative stress and other functions [16, 47, 48]. Xi et al. uncovered that salidroside could promote SOCS5 transcription to reduce airway inflammation and airway remodeling in asthmatic mice [48]. Tsai et al. also proved that SOCS5 was highly expressed in high glucose-induced renal mesangial cells, which led to the inactivation of JAK2/STAT3 pathway [49]. Wang el al also reported that inhibition of SOCS5 led to the apoptosis and inflammation in lung epithelial cells by activation of JAK2/STAT3 pathway [50]. It is well known that the JAK2/STAT3 pathway, as a key intracellular signaling pathway, has also been proved to be involved in the progression of COPD disease through inflammation, oxidative stress and apoptosis [51, 52]. A recent study showed that inhibiting SOCS5 significantly promoted cigarette extract-induced inflammatory responses, exacerbating COPD progression [17]. It also shown that maintaining SOCS5 expression could reduce LPS-induced oxidative stress and inflammation and improve myocardial injury [16]. Here, we established a COPD mouse model, whose results further verified that overexpression of SOCS5 inhibited JAK2/STAT3 pathway activation, improved lung function and reduced oxidative stress in COPD mice. The above reports further confirm our conjecture that SOCS5/JAK2/STAT3 signaling may be involved in the occurrence and development of COPD by influencing oxidative stress levels. Besides, the alteration of SOCS5 in diseases was confirmed to be related various regulation such as miRNAs sponge, DNA methylation, m6A modification and transcription regulation [46, 48, 53], and these regulatory pathways were acted an important role in COPD pathogenesis [54–56]. Thus, it was implied that the exact mechanism of abnormally expressed SOCS5 in COPD was worthy of research.

## Conclusions

In conclusion, our results suggest that sequential NIPPV + IMT treatment alleviated dyspnea and improved exercise tolerance and quality of life in COPD patients, which might be related to reduced levels of oxidative stress mediated by SOCS5/JAK2/STAT3 signaling pathway. Our proposed a new and more effective therapeutic strategy for COPD patients and elucidated its potential pathways of action, providing a direction for the clinical treatment of patients with COPD.

### Electronic supplementary material

Below is the link to the electronic supplementary material.

Supplementary Material 1

Supplementary Material 2

Supplementary Material 3

## Data Availability

All data generated or analyzed during this study are included in this published article.

## Abbreviations

COPD: Chronic obstructive pulmonary disease
NIPPV: Noninvasive positive pressure ventilation
IMT: Inspiratory muscle training
OT: Oxygen therapy
SOCS5: Suppressor of cytokine signaling 5
STAT: Signal transducer and activator of transcription
SSI: STAT inhibitor
JAK2: Janus kinase 2
6MWT: 6-minute walking test
ATS: American Thoracic Society
SRI: Severe respiratory insufficiency
ROS: Reactive oxygen species
MDA: Malondialdehyde
SOD: Superoxide dismutase
GSH: Glutathione
qRT-PCR: Quantitative real-time polymerase chain reaction
