# Supplementary material for: Sequential inspiratory muscle exercise-noninvasive positive pressure ventilation alleviates oxidative stress in COPD by mediating SOCS5/JAK2/STAT3 pathway
Source: BMC Pulm Med. 2023 Oct 12;23:385. doi: 10.1186/s12890-023-02656-5 (PMC10568888; doi:10.1186/s12890-023-02656-5)
Supplement: Supplementary file 1 — Supplementary Material 1 [file 12890_2023_2656_MOESM1_ESM.docx]

**Supplementary Table 1. 6-min walking distance registration form**

| Basic  information | Name |  | | | Random number | |  | |
| --- | --- | --- | --- | --- | --- | --- | --- | --- |
|  | Sex |  | | | Height | |  | |
|  | Age |  | | | Weight | |  | |
| Current diagnosis |  | | | | | | | |
| Pulmonary function classification |  | | | | | | | |
| Take medicine | Name |  | | | Dosage | |  | |
|  | Time | Heart rate | Breathe | Blood pressure | | Borg  dyspnea score | | SpO_2_ |
| Start testing |  |  |  |  | |  | |  |
| End of Test |  |  |  |  | |  | |  |
| Walking distance | Total distance: Time × 30 m＋ m = m | | | | | | | |
| Symptoms of patients during the trial |  | | | | | | | |
| Notes | Whether there is a pause or stop in the middle:  Other: | | | | | | | |
