# Supplementary material for: Sequential inspiratory muscle exercise-noninvasive positive pressure ventilation alleviates oxidative stress in COPD by mediating SOCS5/JAK2/STAT3 pathway
Source: BMC Pulm Med. 2023 Oct 12;23:385. doi: 10.1186/s12890-023-02656-5 (PMC10568888; doi:10.1186/s12890-023-02656-5)
Supplement: Supplementary file 2 — Supplementary Material 2 [file 12890_2023_2656_MOESM2_ESM.docx]

**Supplementary Table 2. Borg rating scale**

| 0 | Not at all |
| --- | --- |
| 0.5 | Very slight, almost unnoticed |
| 1 | Very slight |
| 2 | Mild |
| 3 | Moderate |
| 4 | Some serious |
| 5 | Serious |
| 6 |  |
| 7 | Very serious |
| 8 |  |
| 9 |  |
| 10 | Very serious (maximum) |
