# Supplementary figures and images for: Sequential inspiratory muscle exercise-noninvasive positive pressure ventilation alleviates oxidative stress in COPD by mediating SOCS5/JAK2/STAT3 pathway
Source: BMC Pulm Med. 2023 Oct 12;23:385. doi: 10.1186/s12890-023-02656-5 (PMC10568888; doi:10.1186/s12890-023-02656-5)

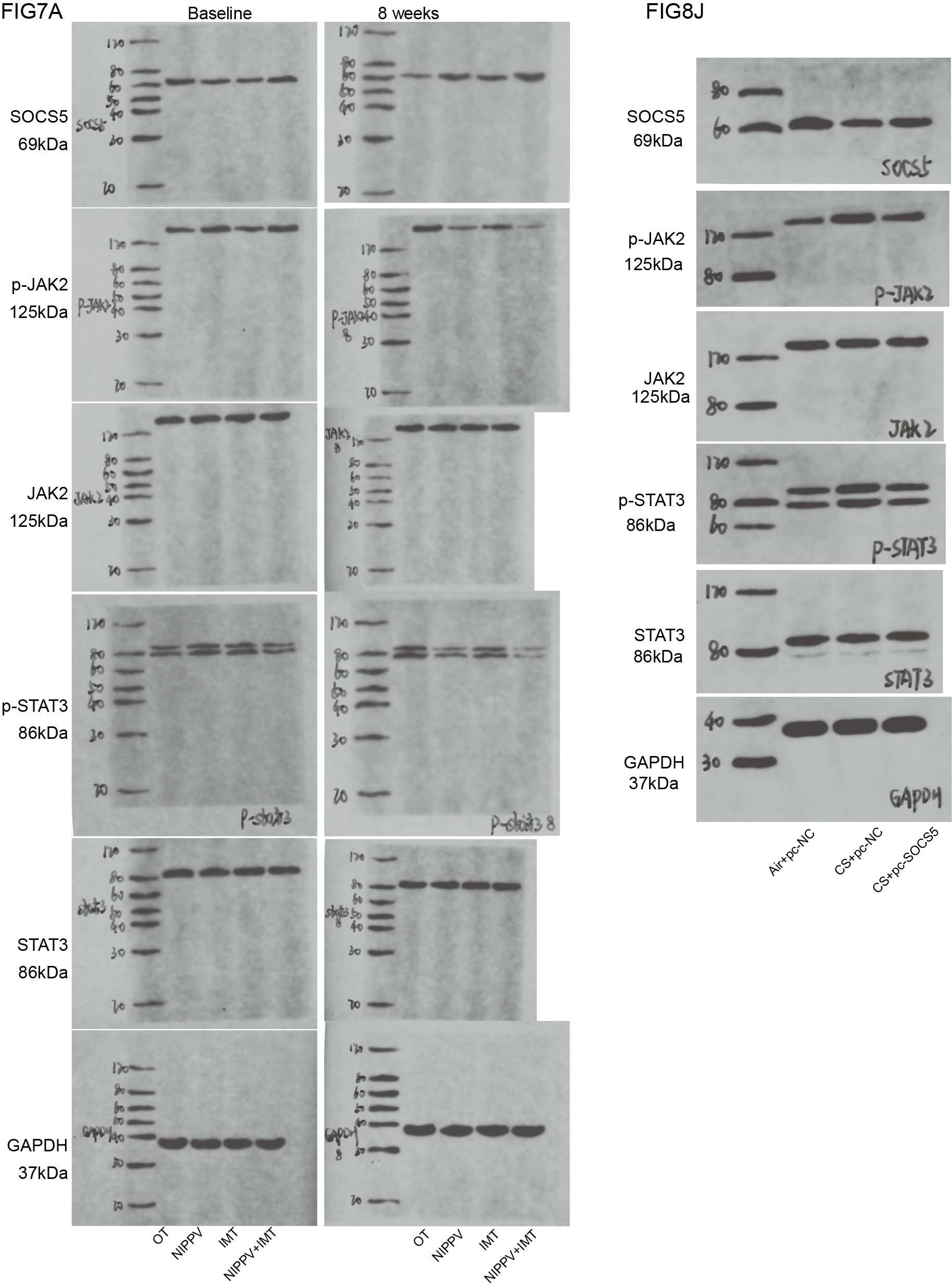

Supplement: Supplementary file 3 — Supplementary Material 3 [file 12890_2023_2656_MOESM3_ESM.tif]
